# Implant loss and crestal bone loss in early loading versus delayed and immediate loading in edentulous mandibles. A systematic review and meta-analysis

**DOI:** 10.4317/jced.57966

**Published:** 2021-04-01

**Authors:** Beatriz Pardal-Peláez, Javier Flores-Fraile, José-Luis Pardal-Refoyo, Javier Montero

**Affiliations:** 1 DDS, PhD. Associate professor. Faculty of Medicine. Dental Clinic. Department of surgery. Alfonso X St. 37007. University of Salamanca. Salamanca. Spain; 2 MD, PhD. Honorary collaborating professor. Faculty of Medicine. IBSAL (Salamanca Biomedical Research Institute) member. University assistance complex of Salamanca. Salamanca. Spain; 3 DDS, PhD. Lecturer of Stomatological Prosthesis. Faculty of Medicine. Dental Clinic. Department of surgery. Alfonso X St. 37007. University of Salamanca. Salamanca. Spain

## Abstract

**Background:**

Information about the risk of early loss and crestal bone loss of dental implants which have been loaded early is scant if compared with data available for those conventionally or immediately loaded. A meta-analysis of early loss and crestal bone loss in immediate or delayed loaded full mandibular denture retaining dental implants has been recently published. It is interesting to evaluate also the risks of early versus immediate and delayed loading in complete mandibular restorations. The purpose of this systematic review and meta-analysis was to study early (EL) versus immediate (IL) and delayed loading (DL) protocols in edentulous mandibles to determine whether differences exist in implant success and crestal bone loss.

**Material and Methods:**

The literature review was conducted in PubMed, Web of Science, and the Cochrane Library. Seven randomized clinical trials were included.

**Results:**

The result of a meta-analysis of implant loss before 1 year in EL versus IL was 0.34 (95% CI: 0.08, 1.52), favoring the EL control group, while the outcome for crestal bone loss at the three-year observation was -0.10 (95% CI: -0.28, 0.09), with a tendency toward reduced bone loss for EL. In the EL versus DL group, the result of the meta-analysis of implant loss before one year was inconclusive, while in the comparison regarding crestal bone loss in the first year of observation, the result was -0.03 (95% CI: -0.08,-0.02) with a tendency to less bone loss in EL.

**Conclusions:**

The risk of early implant loss in the IL group was higher than in the EL group. The results in terms of early implant loss in EL versus DL are inconclusive. Besides, crestal bone loss is greater in immediately and delayed loaded implants, at 1 and 3 years of observation, compared to those loaded early.

** Key words:**Dental implants, early dental implant loading, dental prostheses, implant- supported, alveolar bone loss, meta-analysis.

## Introduction

At the end of the 1970s, there was a paradigm shift in both complete and partial oral rehabilitation thanks to the introduction of dental implants, together with the concept of osseointegration (1). The three options for the rehabilitation of the edentulous mandible (removable complete prosthesis, overdenture and fixed prosthesis on implants) involve a cost range from the most economical to the most expensive. This is due to the fact that implant-borne fixed prostheses require greater prosthodontic resources and a greater number of implants. It is now known that there is no single standard of treatment for the edentulous mandible, as there is evidence that results in terms of patient’s satisfaction and comfort depend more on the decision of the properly informed patient than on the clinical decision of the operator (2).

Furthermore, the period from tooth loss to rehabilitation with implants is perceived by patients as incapacitating and traumatic. In addition, conventional removable mandibular prostheses present instability and lack of retention that compromise the function, aesthetics (3,4) and, ultimately, the quality of life of the patient (5). Shortening the loading periods of implants in edentulous mandibles is beneficial. For this reason, there is a tendency in implantology to reduce treatment times in order to increase patient satisfaction (4).

In the 1990s it was demonstrated that osseointegration can be achieved satisfactorily with immediate or early loading protocols (6,7).

In 2008, at the ITI Consensus Conference, the following definitions were established for the different loading protocols (8): a- conventional or delayed loading: the loading of implants more than two months after their placement; b- early loading: the loading performed between 1 week and 2 months after implant placement; and c- immediate loading: the loading performed within the first week after implant placement.

The aim of this systematic review was to study early loading of dental implants and compare it with delayed loading and immediate loading in edentulous mandibles, and to establish whether there are differences in implant success (which is measured by observing failure by the first year of function and crestal bone loss). This work arises as a complement to a work previously published by this same study group in which a comparative study was carried out in the form of a systematic review with a meta-analysis of immediate loading versus delayed loading in edentulous mandibles (9).

## Material and Methods

The following Patients, Interventions, Comparison, Outcome question was raised:

In patients requiring mandibular complete implant restoration by means of fixed or removable prosthesis, is the loading protocol (immediate, early, or delayed) determinant in terms of implant loss during the first year after implant loading and crestal bone loss of the implants considered as surviving?

Randomized clinical trials carried out with total mandibular edentulous human patients, regardless of the type of prosthesis or full dentition in the maxillary arch, were included, both in English and in Spanish. In the works selected, early loading versus immediate and delayed loading were compared, and both root-shaped implants placed in mature bone and implants placed in a conventional manner or by guided surgery were considered. These could have been performed with or without a flap, with a minimum follow-up of one year.

Clinical cases and non-randomized retrospective or prospective studies, together with those without a control group, were excluded. Randomized clinical trials which compare immediate vs. conventional loading were also excluded, as we have recently published a systematic review and meta-analysis on this topic (9). Similarly, studies that compared either the same type of loading with different implant brands, or implants placed immediately after extraction were not considered, as well as areas that required previous bone grafts (autografts or xenografts) or simultaneous bone grafts. Zygomatic implants were also excluded.

With regard to the literature search, it was conducted in PubMed, Cochrane Library, and Web of Science by using the following terms: ((((((((total edentulous) OR total tooth loss) OR mouth, edentulous(MeSH Terms)) OR jaws, edentulous(MeSH Terms))) AND dental implants)) AND (immediate dental implant loading OR early dental implant loading))) AND ((English(Language)) OR Spanish(Language)))) filtered by randomized controlled trial in PubMed. As for Cochrane Library, search was filtered by trial with the terms ((total edentulous OR total tooth loss OR mouth, edentulous OR jaws, edentulous) AND dental implants) AND (immediate dental implant loading OR early dental implant loading). Finally, ((total edentulous OR total tooth loss OR mouth, edentulous OR jaws, edentulous) AND dental implants) AND (immediate dental implant loading OR early dental implant loading) refined by clinical trial were used in Web of Science. These 3 searches offered a total of 691 records. Eight additional articles were identified by means of other sources, which we included as related articles. After duplicates had been discarded, the sample consisted of a total of 406 articles, 11 of which were read in detail: those whose title and summary matched the inclusion and exclusion criteria.

Two papers were excluded (10,11) because they had the same sample as 2 other studies that were selected and had a longer observation period (12,13). Another study was excluded because the patients were not randomized but treated consecutively (14). The randomized clinical trial by Cannizaro *et al* (15) was excluded because, despite having a correct design and a low risk of bias, it included implants placed in post-extraction alveoli.

Seven studies were considered for the qualitative and quantitative synthesis. Five of them were included in the early loading vs. delayed loading comparison and 2 in the early loading vs. immediate loading group. With regard to timing, there was no time limitation. The last search was made on April 27, 2020.

**Figure 1:**
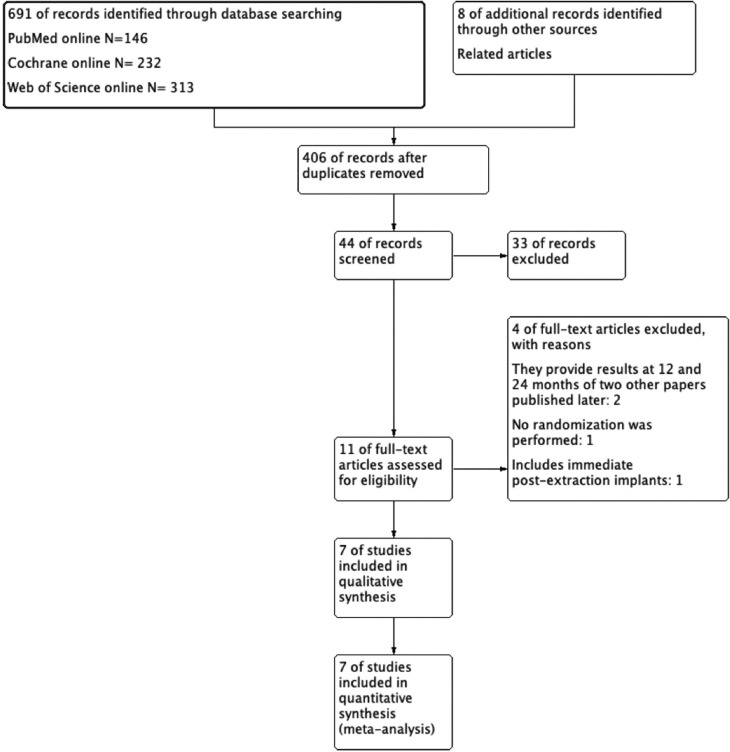
Flow chart of included studies.

Figure 1 includes details about the search strategy, which is specified in the flow chart following the PRISMA statement (16).

The meta-analysis was carried out using a software program (Revman; Review Manager, v5.3, Copenhagen: The Nordic Cochrane Centre; The Cochrane Collaboration, 2014). The odds ratio (OR) was used for the dichotomous variables and the difference of means (DM) for the continuous variables. The 95% confidence interval (CI) (α=.05) was estimated.

Heterogeneity was estimated by inspection of the forest-plot (through overlapping confidence intervals) and estimation of I2 and Chi-square. A sensitivity analysis was performed excluding one of the studies each time to identify possible changes in the results.

Two comparison groups and two independent analyses were established: a- early loading (EL) versus immediate loading (IL); and b- early loading (EL) versus delayed loading (DL). A descriptive analysis was performed in all comparisons, and, where possible, a quantitative analysis (meta-analysis) was also carried out.

The risk of bias of each study was performed following the Cochrane tool for the assessment of risk of bias of randomized clinical trials (17). Outcome quality was assessed through the GRADE classification system (18) and the GRADEpro 3.2 software program (19); the studies were therefore assigned an appropriate level of evidence.

Two researchers (BP and JLP) assessed the risk of bias and the quality of the papers, and discrepancies were resolved by discussion.

## Results

Seven randomized clinical trials comparing early versus delayed loading (5 trials) and early versus immediate loading (2 trials) in total mandibular edentulous patients, regardless of the type of dentition or prosthesis in the maxillary arch, were selected.

The minimum follow-up time established as an inclusion criterion was 12 months; therefore, the observation period ranged from 12 to 84 months.

The total number of implants placed was 500, of which 138 were conventionally loaded, 112 were immediately loaded and 250 were early loaded.

The type of prosthesis used was the overdenture on 2 (12,13,20-22) or 4 implants (23).

Most of the papers studied non-splinted implants using ball attachments (12,13,22–24). Although Payne *et al*’s (21) work does not specify the type of attachment used, it does indicate that the two implants used were not splinted. The Dolder bar was used for implant splinting (25).

Implants with standard widths between 3.3 and 4.1 mm and an estimated ideal length of more than 10 mm were used in most studies.

Only 1 work established that the minimum torque to consider immediate or early loading as suitable was of 35 Ncm; the rest of the papers did not specify this parameter (20). Two papers provided RFA data using Osstell (Integration Diagnostics, Gothenburg, Sweden) (12,13) and another used the Periotest to establish the initial stability of the implants and compare it with the stability at the end of the observation time (22).

In relation to the measurement of predictive parameters for implant survival and success, vertical crestal bone loss was considered a determining measurement. All works provided quantitative data of bone loss in millimeters at the end of the study period.

Table 1 shows the summary of some of the data collected from the different clinical trials included in this work.

**Table 1:**
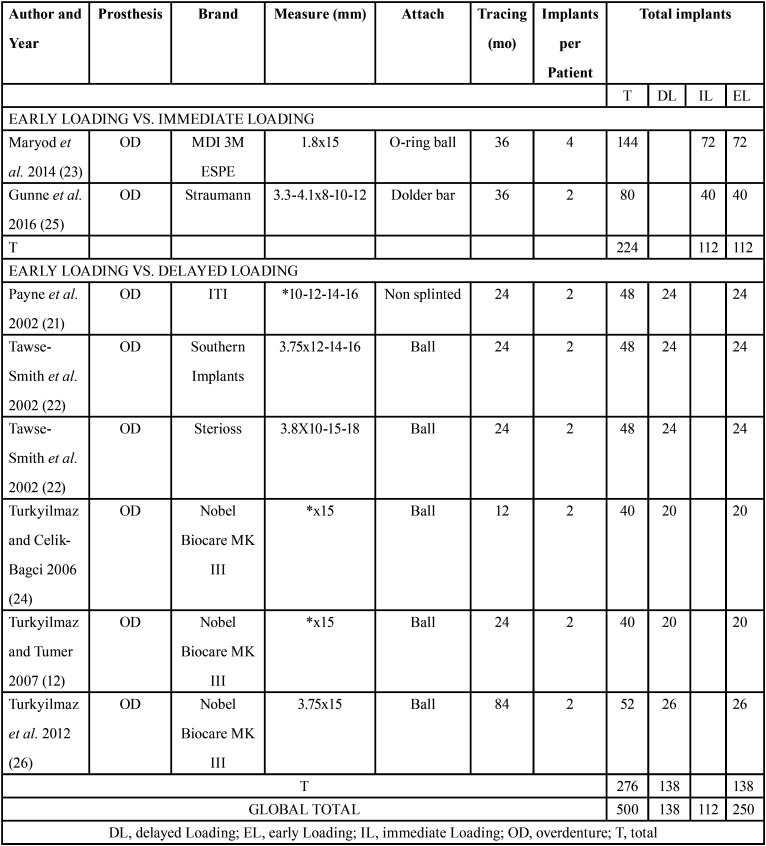
Data collected from the works included.

**Figure 2:**
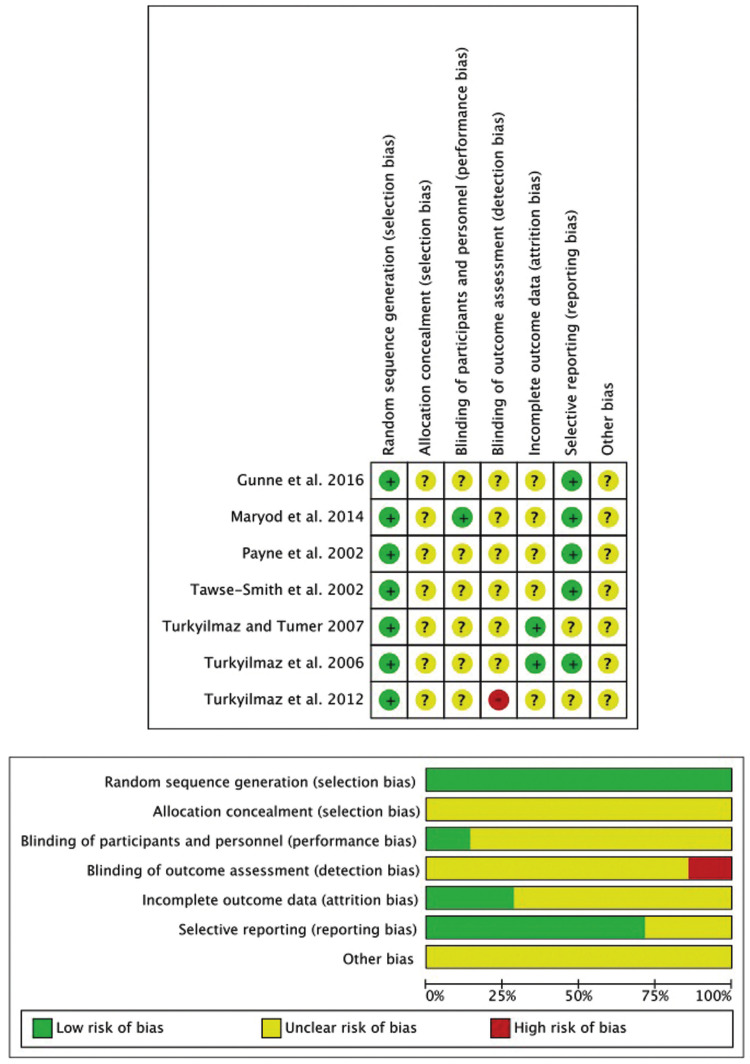
QUADAS-2 graph and summary of the risk of bias of the selected studies.

Figure 2 shows the graph and the summary of the risk of bias in the two comparative groups (early loading versus immediate loading, and early loading versus delayed loading). According to the Cochrane handbook, bias among the studies in this systematic review is classified as unclear, since in all the comparative groups most of the evidence comes from work with a low or unclear risk of bias.

In the early loading versus immediate loading comparison group, 2 papers were selected that met the inclusion criteria for meta-analysis.

**Figure 3:**
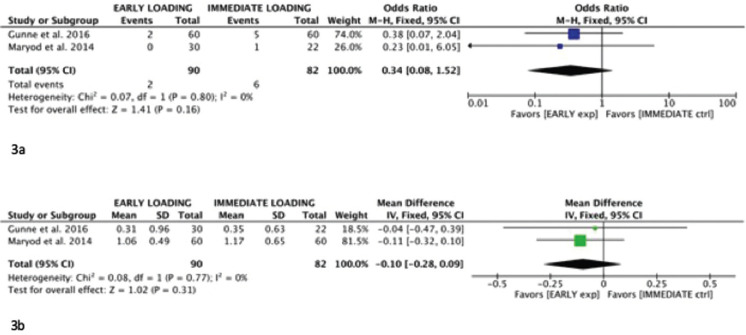
Early failure meta-analysis (3a) and crestal bone loss meta-analysis (3b) (early loading vs. immediate loading).

The heterogeneity between both works was low (I2 = 0%); for this reason, a fixed-effect model was selected (Fig. 3a). There were no graphic or statistical signs of publication bias on the funnel plot.

The meta-analysis showed that, in terms of early failures, early loading is more convenient than immediate loading, as it results in less implant losses before one year (odds ratio: 0.34 (fixed effects 95% CI: 0.08, 1.52)). However, more studies are needed to confirm this tendency.

In relation to crestal bone loss, a fixed-effect model was also chosen (Fig. 3b). In both cases, bone loss at 3 years was assessed, as the papers did not provide information on shorter periods of time. There was no difference in marginal bone loss in immediately loaded versus early loaded implants, although both studies indicated less loss in early loading (mean difference: -0.10 (fixed effects 95% CI: -0.28, 0.09)).

In the early versus delayed loading comparison group, 5 papers were selected. Three of the papers had zero implant failures in both study groups; therefore, they were not estimable for meta-analysis and only 2 papers remained available for analysis.

**Figure 4:**
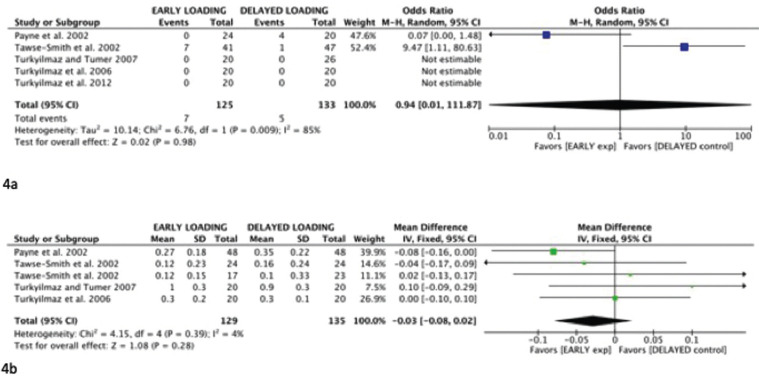
Early failure meta-analysis (4a) and Crestal bone loss meta-analysis at one year (4b) (early loading vs. delayed loading).

Heterogeneity between both works was high (Chi-square=6.76; I2 = 85%). With such a high value of I2, a random effects model was considered, although the usefulness of meta-analysis is considered to be void. The confidence interval is very wide (odds ratio: 0.94 (random effects 95% CI: 0.01,111.87)), which indicates that more and better designed works are needed to make meta-analysis useful (Fig. 4a).

With regard to crestal bone loss during the first year, the I2 was 4%. For this reason, a fixed effect model was selected, the meta-analysis shows a trend towards less bone loss in early loading, but no difference between groups (mean difference: -0.03 (fixed effects 95% CI: -0.08, 0.02)) (Fig. 4b). The funnel plot showed no publication bias.

## Discussion

A total of 7 randomized clinical trials were selected in this work, of which 2 compared early versus immediate loading and 5 compared early versus delayed loading. Two independent analyses were conducted for each of the comparison groups.

The risk of bias of studies comparing early versus immediate loading (23,25) and those comparing early versus delayed loading (12,13,21,22,24) is considered unclear. However, in general, these trials are less well designed than those in the previously published immediate versus delayed loading comparative block (9), particularly in terms of randomization sequences and blinding of operators and observers. It should be noted that, in most studies analyzed, the authors omitted information concerning the concealment of the sequence of randomization and blinding of operators and assessors, which has led to the risk of bias in these parameters being assessed as unclear, when perhaps it should be considered as high risk, if this lack of information is taken as a lack of blinding.

Early implant loss was assessed by 2 different meta-analyses depending on the study groups. The results taking as effect size index the Odds Ratio (95% CI) were, for the comparisons between early versus immediate loading and early versus delayed loading, of 0.34 (0.08, 1.52) and 0.94 (0.01, 111.87), respectively. Therefore, early loading is preferred to immediate loading. The confidence interval in terms of early failures in the early versus delayed loading comparison is so wide that it is not possible to establish which protocol is better. More studies are needed to estimate the effect.

In all studies comparing early versus delayed and immediate loading, crestal bone loss around the implant is assessed using calibrated, parallel periapical radiographs and image processing software (12,13,21-25). Nevertheless, according to Walton and Layton (27), crestal bone loss measurements showing a difference of 1 mm or less can be attributed to limitations in measurement rather than biological factors. Although most studies selected evaluate crestal bone loss as a key element in determining implant success, there are differences in the length of observation times for each study, ranging from 12 to 84 months. It seems logical to think that the crestal bone loss that can be related to the type of loading that the implant received is the one measured within the first year of function; for this reason, additional analyses were performed at one year of observation when possible. In the studies comparing early and delayed loading (12,13,21,22,24), a subgroup was created to evaluate crestal bone loss at one year of observation (12,21,22,24). It was taken into account that in the study by Tawse-Smith *et al.* (22) there are two different comparisons when comparing implants of two different brands and they were counted as two different studies. Only one paper provided no information on marginal bone loss after one year (13). The results at one year (mean difference -0.03 (fixed effects,95% CI: -0.08, 0.02)) they favored early loading. Besides, neither of the 2 studies comparing early versus immediate loading (23,25) provided data on crestal bone loss after one year, so the loss was analyzed at the end of the study period (3 years); the value (mean difference: -0.10 (fixed effects 95% CI: -0.28, 0.09)) also favored early loading. More studies are needed to determine which type of loading causes less crestal bone loss.

With regard to the comparison of failures of splinted or non-splinted implants, the comparison could just be conducted in the early versus immediate loading group, since in the early versus delayed loading group only non-splinted attachments were used. In the immediate versus early loading group, no difference was found between non-splinted (23) and Dolder bar splinted implants (25).

Table 2 assesses the quality of evidence and the degree of recommendation of each method of the studies comparing early versus immediate loading. The selected papers offered a high recommendation level for early implant loss and moderate for crestal bone loss at 3 years; thus, early loading is recommended instead of immediate loading.

**Table 2:**
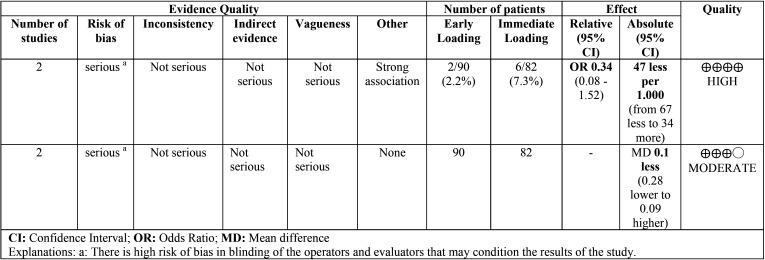
Quality of the evidence (Early loading vs. immediate loading).

Table 3 assesses the quality of evidence and the degree of recommendation of the clinical trials comparing early loading with delayed loading. A very low level of evidence was obtained for early implant loss and a moderate level was obtained for bone loss in favor of conventional loading. Conventional loading is suggested instead of early loading.

**Table 3:**
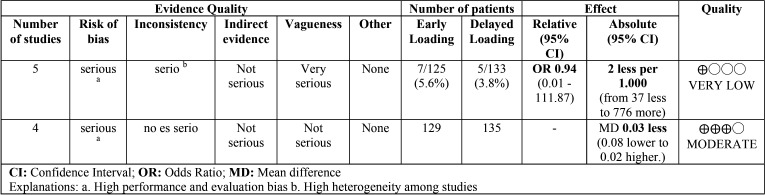
Quality of the evidence (Early loading vs. delayed loading).

The limitations of this systematic review are mainly due to the low number of randomized clinical trials comparing loading protocols, shortcomings in the designs, heterogeneity in terms of implant observation times, and the lack of assessment of marginal bone loss in some studies. An adequate measurement of the primary stability of the placed implants as well as the objective evaluation of the bone quality in which they are placed would be desirable.

It is also important to note that this work has not taken into account the patient’s quality of life, which is an important factor to be considered when making clinical decisions.

## Conclusions

1. The risk of implant loss before 1 year after loading is higher in immediate loading implants as compared to early loading implants.

2. The risk of implant loss during the first year between early loading and delayed loading cannot be assessed.

3. There are no differences between immediate loading versus early or versus delayed loading in terms of crestal bone loss. In general, when early loading is performed, bone loss tends to be less between 1 and 3 years after implant loading.

4. More studies are needed to make firm recommendations on early loading protocols, although the quality of the available evidence recommends early rather than immediate loading, and delayed rather than early loading.
